# Epidemiological analysis of pediatric respiratory pathogens in Hunan, China: a retrospective multicenter study from 2022 to 2024

**DOI:** 10.1186/s12879-025-12283-6

**Published:** 2025-12-10

**Authors:** Si-Jing Long, Rong Hu, Jian-Ping Ouyang, Chang-Jun Tian, Tai-Yong Guo, Qi-Lin Luo, Xiang-Fu Liao, Rong Yang, Di-Jun Liu, Tao Liu, Yong-Hong He, De-Jun Wu, Zhang He, Guo-Feng Zou, Ji-Zhi Xu, Dong-Fang Lei, Li-Qiong Yang, Yan-Ping Shen, Yuan-Feng She, Xiao-Yang Zhao, Xiang-Jian Xiang, Xi-Rong Fu, Yuan-Xiang Ou, Ning Yin, Sai-Jun Yi, Jian-Jun Jiang, Li-Li Zhong, Jing-Jing Zhang, Yan Yu

**Affiliations:** 1 Changsha KingMed Center for Clinical Laboratory, Changsha, 410000 China; 2 Department of Pediatric, The First People’s Hospital of Changde City, Changde, 415000 China; 3 Department of Pediatric, Yueyang Maternal and Child Health Care Hospital, Yueyang, 414000 China; 4 Department of Pediatric, Zhangjiajie City People’s Hospital, Zhangjiajie, 427000 China; 5 Department of Pediatric, The Third People’s Hospital of Yongzhou, Yongzhou, 425000 China; 6 Department of Pediatric, Hunan University of Medicine General Hospital, Huaihua, 418000 China; 7 Department of Pediatric, Yueyang Central Hospital, Yueyang, 414000 China; 8 Department of Pediatric, Yuanling County People’s Hospital, Xiangxi, 416000 China; 9 Department of Pediatric, Yueyang People’s Hospital, Yueyang, 414000 China; 10 Department of Pediatric, Yiyang Central Hospital, Yiyang, 413000 China; 11 Department of Pediatric, Guiyang County Maternal and Child Health Care Hospital, Chenzhou, 423000 China; 12 Department of Pediatric, Longshan County People’s Hospital, Xiangxi, 416000 China; 13 Department of Pediatric, Lixian People’s Hospital, Changde, 415000 China; 14 Department of Pediatric, Huitong County People’s Hospital, Huaihua, 418000 China; 15 Department of Pediatric, Changsha Maternal and Child Health Care Hospital, Changsha, 410000 China; 16 Department of Pediatric, Yueyang Hospital of Traditional Chinese Medicine, Yueyang, 414000 China; 17 Department of Pediatric, The Central Hospital of Yongzhou, Yongzhou, 425000 China; 18 Department of Pediatric, Shaodong People’s Hospital, Shaodong, 422800 China; 19 Department of Pediatric, The Second People’s Hospital of Huaihua, Huaihua, 418000 China; 20 Department of Pediatric, The First People’s Hospital of Pingjiang, Yueyang, 414000 China; 21 Department of Pediatric, Anren County People’s Hospital, Chenzhou, 423000 China; 22 Department of Pediatric, Youxian People’s Hospital, Zhuzhou, 412000 China; 23 Department of Pediatric, The Third Hospital of Changsha, Changsha, 410000 China; 24 Department of Pediatric, Hengyang Maternal and Child Health Care Hospital, Hengyang, 421000 China; 25 Department of Pediatric, The People’s Hospital of Louxing District, Loudi, 417000 China; 26 Department of Pediatric, Qiyang People’s Hospital, Qiyang, 426100 China; 27 Hunan Provincial Key Laboratory of Pediatric Respirology, Hunan Provincial People’s Hospital (The First Affiliated Hospital of Hunan Normal University), Changsha, 410000 China; 28 Key Laboratory of Molecular Epidemiology of Hunan Province, School of Medicine, Hunan Normal University, Changsha, 410000 China

**Keywords:** Children, Respiratory infection, Hunan, Virus, Targeted next-generation sequencing technology

## Abstract

**Background:**

The zero-COVID policy and non-pharmaceutical interventions (NPIs) implemented in response to COVID-19 concurrently influenced the transmission of other respiratory pathogens. In the post-pandemic era following the policy’s cessation, whether the epidemiological patterns of these respiratory pathogens have reverted to pre-pandemic baselines or persisted unchanged remains a subject of interest.

**Methods:**

The retrospective study analyzed 8,774 cases with acute respiratory tract infections (ARTIs) from 2022 to 2024, with data sourced from 26 children’s hospitals or general hospitals in Hunan Province, to investigate the potential impacts of policy changes on epidemiology. Nine common respiratory pathogens were detected using targeted next-generation sequencing (tNGS) technology.

**Results:**

Among children seeking medical care, the overall pathogen positive rate increased (*P* < 0.001) in both 2023 and 2024, primarily affecting children aged ≤ 1 year and those ≥ 6 years. The positivity rate of respiratory syncytial virus (RSV, both RSV-A and RSV-B) in children aged ≤ 3 years increased and resumed its winter-spring seasonality in 2023–2024, with RSV-A predominant in 2023 and RSV-B in 2024. Human metapneumovirus (HMPV) showing a counter-seasonal pattern. The seasonal epidemics of human parainfluenza virus (HPIV) (mainly HPIV-3) and human bocavirus (HBoV) occur earlier in summer. Influenza A virus (FluA) established typical circulation pattern with influenza B virus (FluB) in autumn and winter. The positive rate of single infection and co-infection of pathogens increased (*P* < 0.01). The negative co-occurrence patterns between viruses are common. The restricted cubic spline (RCS) model indicates that the susceptible populations for RSV, HMPV, HPIV, and FluA have changed.

**Conclusion:**

With the end of the Zero-COVID policy, some respiratory pathogens, including RSV, HPIV, HBoV, and Flu, show clear seasonality. Clinics should closely monitor the epidemiological trends of pediatric respiratory pathogens in the post-pandemic period.

**Clinical trial number:**

Not applicable. This study is a retrospective analysis based on clinical data. Since it only involves statistical analysis and no clinical trials, a clinical trial number is not required.

**Supplementary Information:**

The online version contains supplementary material available at 10.1186/s12879-025-12283-6.

## Introduction

Acute respiratory tract infections (ARTIs) are one of the main causes of hospitalization in children, posing a serious threat to children’s health, with the characteristics of recurrent attacks and long course of illness [1]. Viruses are common pathogens causing ARTIs in children. For example, in a surveillance in China from 2009 to 2019, it was found that the positive rate of viruses in children under 5 years old and preschool children reached 46.9%, among which RSV (Respiratory syncytial virus) had the highest positive rate [2], RSV is also the main pathogen causing bronchiolitis in infants [3]. In addition to RSV, common viruses causing ARTIs in children also include human rhinovirus (HRV), human adenovirus (HAdV), human parainfluenza virus (HPIV), human metapneumovirus (HMPV), human bocavirus (HBoV), influenza A virus (FluA), influenza B virus (FluB), and human coronavirus OC43 (HCoV-OC43). ARTIs can be caused by one or more viral infections. Due to their relatively weak immune system, children who are infected with multiple pathogens have a higher probability of developing severe and critical conditions.

The emergence of SARS-CoV-2 in 2019, which led to the COVID-19 pandemic, has greatly affected people’s normal lives. Therefore, various non-pharmaceutical interventions (NPIs) were implemented nationwide, including urging the public to reduce social interactions, wear masks, suspend in-person classes, strengthen online education, and close borders. The purpose of these measures was to slow down the spread of SARS-CoV-2, alleviate the demand for medical resources, and buy time for vaccine development and treatment methods. These measures also had an impact on the seasonal circulation patterns of various respiratory pathogens, including RSV and Flu [4]. In December 2022, China ended its zero-COVID policy and all NPIs were lifted [5], however, the impact of this on the spread of other ARTIs-related pathogens in Hunan region is still unclear. The prevalence and pathogen spectrum of ARTIs-related pathogens vary in different countries, regions, as well as different years and seasons [6–8]. Therefore, timely detection of these pathogens is crucial for the diagnosis, prevention, and control of ARTIs in local children.

Traditional pathogen detection mainly relies on morphological examination, pathogen culture, serology, and other methods. However, these detection methods have problems such as low sensitivity, lengthy procedures, and limited coverage of pathogen types [9–11]. In contrast, molecular diagnostic techniques have significantly improved our ability to identify pathogens. Among them, targeted next-generation sequencing (tNGS), which combines multiplex PCR amplification and next-generation sequencing, can detect dozens to hundreds of known pathogens in a single sample. This technology has shown excellent performance in pathogen detection [12, 13].

This study aims to analyze the epidemiological changes of ARTIs-related pathogens in Hunan region before and after the full relaxation of NPIs and the lifting of the zero-COVID policy, providing direction for the monitoring and prevention of ARTIs-related pathogens in children.

## Methods

### Study population

This study is a multicenter retrospective study based in Hunan Province, a region with a population of over 60 million residents. It was led by Hunan Provincial People’s Hospital (The First Affiliated Hospital of Hunan Normal University) and involved the participation of 26 children’s hospitals or general hospitals across Hunan Province, covering 14 cities and prefectures in the province. The study subjects included patients with ARTIs who visited local hospitals from January 1, 2022 to October 31, 2024. The year 2024 only covers data from the first ten months (January–October) and thus does not represent a complete annual cycle. The inclusion criteria are as follows: (1) The patient is 14 years old or younger. (2) Diagnosed with ARTIs, and/or presenting with the following symptoms or signs: fever, cough, tachypnea, sore throat, rhinorrhea, abnormal lung breath sounds. (3) Having complete clinical data. (4) Children who underwent targeted next-generation sequencing (tNGS) testing. Children with malignant tumors, hematological diseases, history of chemotherapy, tuberculosis, asthma, steroid treatment for more than 30 days, or immunosuppressive therapy were excluded. Cases with missing information on age, gender, or clinical diagnosis were also excluded. Relevant information was obtained through the hospital’s electronic information system.

### Laboratory testing

The nasopharyngeal swab samples of the children were transported to Changsha KingMed Diagnostics Laboratory for testing, using a targeted next-generation sequencing (tNGS) technique for multiple respiratory pathogens. This technology combines multiplex PCR with next-generation sequencing (NGS), targeting the highly conserved regions of the genomes of respiratory pathogens. The detectable pathogens include HRV, RSV (RSV-A, RSV-B), HAdV, HPIV (HPIV-1, HPIV-2, HPIV-3, HPIV-4), HMPV, HBoV, FluA, FluB, and HCoV-OC43. Specific primers are designed for the pathogens, and PCR amplification is performed in a single tube to enrich the target pathogens. In the second round of PCR, sequencing adapters that distinguish the source of the sample are connected. A gene sequencer (KM MiniSeqDx-CN) is used to perform high-throughput sequencing on the PCR products. Bioinformatics software or programs are employed to conduct quality control, filtering, genome alignment, and coverage statistics on the sequencing data. For RSV, HPIV, and Flu subtyping, PCR primers were designed to target species - level specific regions. Subtyping was achieved by comparing the specificity of the amplified products. The bioinformatics analysis results are manually interpreted: the positivity of pathogens is determined by considering factors such as the pathogenicity of the pathogens, the coverage and specificity of the amplicons, and the number of sequences. For viruses, amplicon coverage ≥ 50% with a normalized sequence count ≥ 3, or a normalized sequence count ≥ 10. All results were reviewed by two professionals. tNGS is used for children with severe clinical symptoms or those whose routine RT-PCR test results are negative, or for children suspected of having mixed infections. All the standard protocols established by the laboratory remained consistent throughout the entire study period.

### Statistical analysis

Considering that the zero-COVID policy was lifted in December 2022, we designate 2022 as the pre-lifting period and 2023–2024 as the post-lifting period of the zero-COVID policy.

Statistical analysis was performed using SPSS software, version 29.0 and R software, version 4.4.2. Categorical data were described using counts and percentages. The chi - square test was used for separate comparisons between 2022 and 2023 and between 2022 and 2024. P < 0.05 was considered statistically significant. In this study, Spearman correlation analysis was used to examine the co-occurrence or negative co-occurrence patterns between the two pathogens. Co-occurrence patterns were defined as a tendency for simultaneous infection, while negative co-occurrence patterns were defined as a lower likelihood of concurrent infection. The association between age and pathogen infection risk was analyzed using the restricted cubic spline regression model (RCS), with the data from the same age group in 2022 as the reference group, to analyze the changes in the risk of pathogen infection in different age groups from 2023 to 2024. *P* < 0.05 was considered to indicate statistical significance.

## Results

### Basic characteristics and pathogen positive rate changes of children with ARTIs from 2022 to 2024

**Fig. 1:**
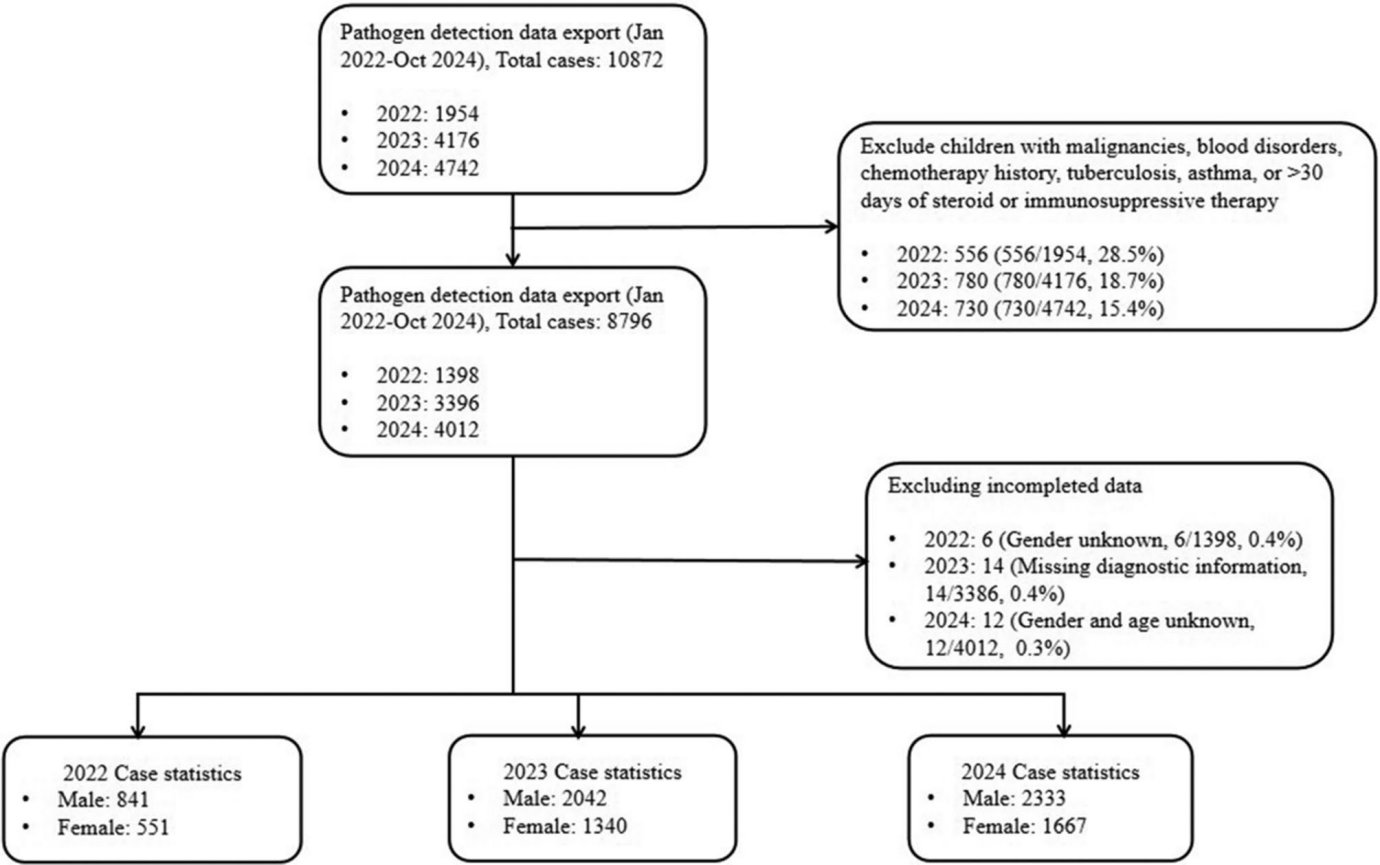
The flowchart of case enrollment

A total of 8,774 children were included, with 1,392 cases in 2022. The number of hospital visits increased in 2023–2024, and according to our predetermined inclusion criteria, 3,382 and 4,000 cases were included in 2023 and 2024, respectively (Fig. 1). From 2022 to 2024, a total of 8774 children were included in the study, with 5216 boys and 3558 girls, resulting in a male-to-female ratio of 1.47:1. The children were divided into five age groups: 0–6 months (1591, 18.1%), 7–12 months (1937, 22.1%), 2–3 years (1805, 20.6%), 4–5 years (1465, 16.7%), and ≥ 6 years (1976, 22.5%). The median age of the children was 2 years in 2022 and 2023, and it increased to 3 years in 2024.

64.1% (5627/8774) of the children tested positive for at least one of the nine pathogens. The positivity rates in 2023 and 2024 were higher than in 2022 (2023: 65.9% vs. 2022: 56.4%, 2024: 65.3% vs. 2022: 56.4%, both *P* < 0.001), with increased disease rates for both among male and female children (*P* < 0.01). In 2022, the highest positivity rate was observed in the 2–3 years age group (71.9%), followed by the 4–5 years (65.6%) and 7–12 months (65.5%) age groups. The positivity rates for the 0–6 months and ≥ 6 years age groups were lower at 41.2% and 40.1%, respectively. In 2023, the positivity rate in the 7–12 months age group increased and was comparable to the 2–3 years age group. Additionally, the positivity rates for the 0–6 months and ≥ 6 years age groups also increased (*P* < 0.001). In 2024, the positivity rates for the 0–6 months, 7–12 months, and ≥ 6 year age groups were still higher than those in 2022 (*P* < 0.001). Specifically, the positivity rate for the 0–6 months age group increased to 66.1%, for the 7–12 months age group to 76.6%, and for the ≥ 6 years age group to 53.1%. Compared to 2022, the positivity rate of hospitalized children increased in both 2023 and 2024 (*P* < 0.001) (Table 1).

**Table 1:** From 2022 to 2024, the basic characteristics and pathogen positivity rate of children with artis were included

Characteristic	2022	2023	2024	χ2(a)	*P*(a)	χ2(b)	*P*(b)	Total
	(*n* = 1392)	(*n* = 3382)	(*n* = 4000)					(*n* = 8774)
All	785/1392 (56.4%)	2229/3382 (65.9%)	2613/4000 (65.3%)	38.351	0.000	35.345	0.000	5627/8774 (64.1%)
Gender								
Male	478/841 (56.8%)	1384/2042 (67.8%)	1573/2333 (67.4%)	31.167	0.000	30.304	0.000	3435/5216 (65.9%)
Female	307/551 (55.7%)	845/1340 (63.1%)	1040/1667 (62.4%)	8.843	0.003	7.727	0.005	2192/3558 (61.6%)
Age								
0 - 6 months	148/359 (41.2%)	343/513 (66.9%)	475/719 (66.1%)	56.418	0.000	60.56	0.000	966/1591 (60.7%)
7–12 months	201/307 (65.5%)	629/845 (74.4%)	601/785 (76.6%)	8.988	0.003	13.912	0.000	1431/1937 (73.9%)
2–3 years	215/299 (71.9%)	570/770 (74.0%)	530/736 (72.0%)	0.496	0.481	0.001	0.973	1315/1805 (72.9%)
4–5 years	128/195 (65.6%)	340/530 (64.2%)	465/740 (62.8%)	0.138	0.71	0.523	0.47	933/1465 (63.7%)
≥ 6 years	93/232 (40.1%)	347/724 (47.9%)	542/1020 (53.1%)	4.349	0.037	12.88	0.000	982/1976 (49.7%)
Infection type								
Single infection	620//1392 (44.5%)	1721/3382 (50.9%)	1975/4000 (49.4%)	15.895	0.000	9.669	0.002	4316/8774 (49.2%)
2 Co-infections	142/1392 (10.2%)	437/3382 (12.9%)	569/4000 (14.2%)	6.847	0.009	14.606	0.000	1148/8774 (13.1%)
3 or 4 Co-infections	23/1392 (1.7%)	71/3382 (2.1%)	69/4000 (1.7%)	1.021	0.312	0.033	0.857	163/8774 (1.9%)
Overall Co-infections	165/1392 (11.9%)	508/3382 (15.0%)	638/4000 (16.0%)	8.169	0.004	13.673	0.000	1311/8774 (14.9)
Case type								
Inpatients	777/1379(56.3%)	2052/3130(65.6%)	2174/3320(65.5%)	34.765	0.000	34.816	0.000	5003/7829(63.9%)

χ2(a) and P(a) denote the comparison between 2022 and 2023, while χ2(b) and P(b) denote the comparison between 2022 and 2024

### Differences in pathogen detection before and after the lifting of NPIs

**Fig. 2:**
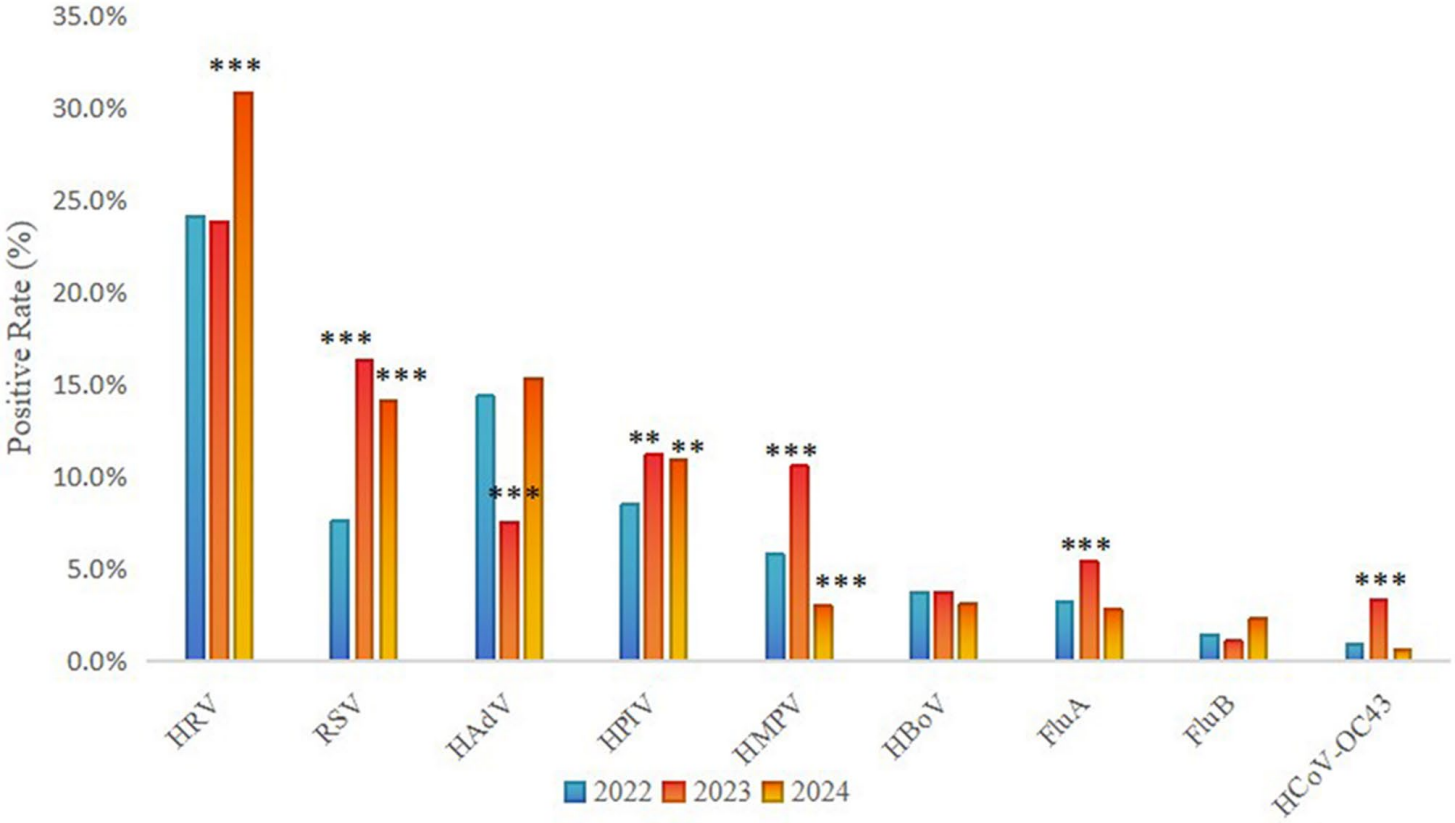
Changes in pathogen positivity rates before and after the lifting of NPIs. Comparisons are made between 2022 and 2023, as well as between 2022 and 2024. *P < 0.05, **P < 0.01, ***P < 0.001. RSV: Respiratory syncytial virus, HRV: human rhinovirus, HAdV: human adenovirus, HPIV: human parainfluenza virus, HMPV: human metapneumovirus, HBoV: human bocavirus, FluA: influenza A virus, FluB: influenza B virus, HCoV-OC43: human coronavirus OC43

After the lifting of NPIs, the positivity rates of several pathogens increased mong the population seeking medical care, including HRV, RSV, HPIV, HMPV, FluA, and HCoV-OC43 (Fig. 2). Specifically, HRV remained the pathogen with the highest positivity rate from 2022 to 2024, with an increase in 2024 (2022: 24.1% vs. 2024: 30.8%, *P* < 0.001). Compared with 2022, the positivity rates of RSV and HPIV both increased in 2023–2024 (RSV *P* < 0.001, HPIV *P* < 0.01), HMPV, FluA, and HCoV-OC43 increased in 2023 (*P* < 0.001), and the HMPV positivity rate decreased from 5.8% in 2022 to 3.0% in 2024 (*P* < 0.001). The positivity rate of HAdV decreased in 2023 (*P* < 0.001). There was no significant difference in the detection rates of HBoV and FluB (*P* > 0.05).

### Epidemiological characteristics of pathogens in children of different age groups

**Fig. 3:**
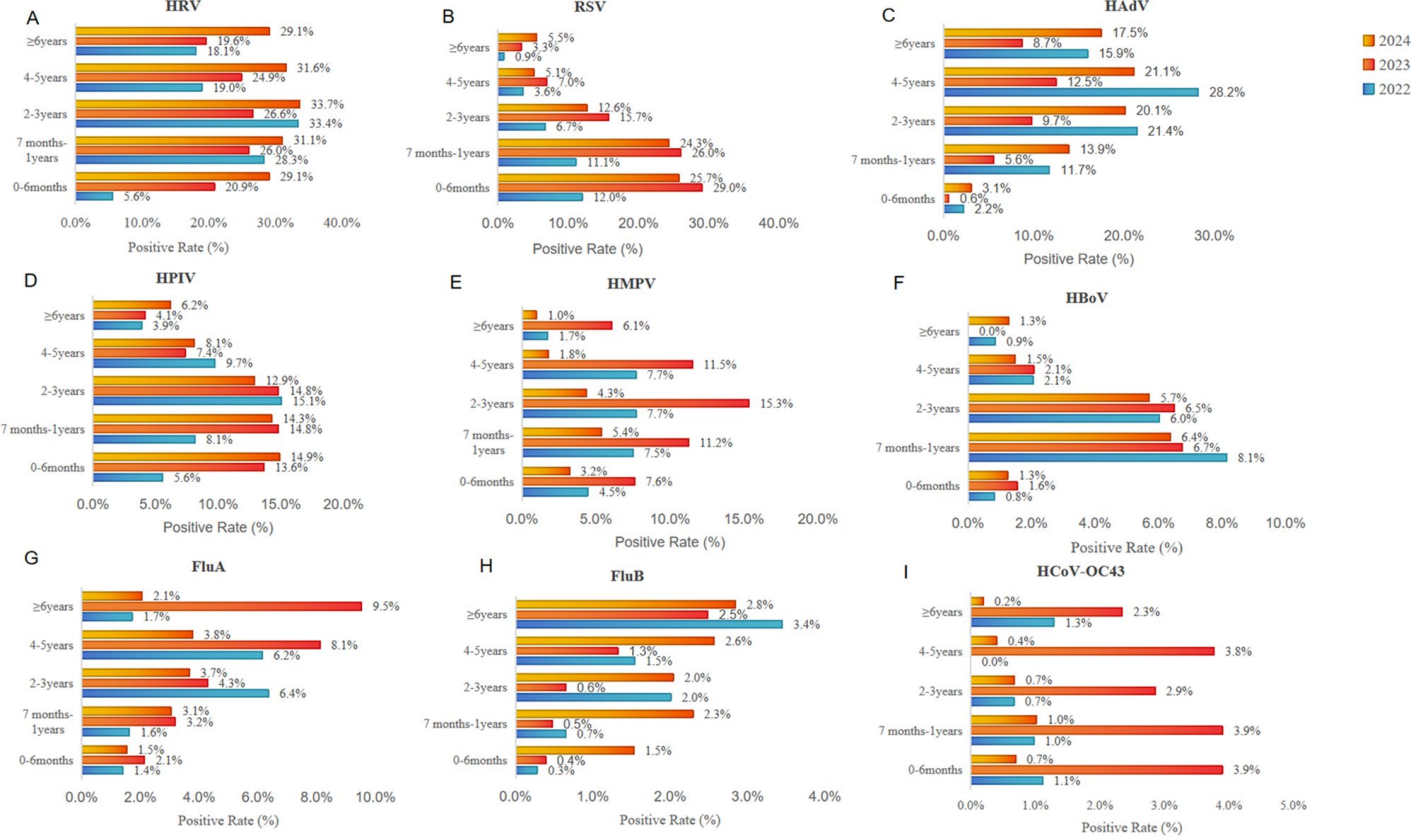
Epidemiological characteristics of respiratory pathogens in children of different age groups from 2022 to 2024. **A**. HRV **B**. RSV **C**. HAdV **D**. HPIV **E**. HMPV **F**. HBoV **G**. FluA **H**. FluB **I**. HCoV - OC43

From 2023 to 2024, among children seeking medical care, the positive rate of HRV in children aged 0–6 months increased significantly, rising from 5.6% in 2022 to 20.9% in 2023 and 29.1% in 2024, which was much higher than in 2022 (*P* < 0.001). The positivity rate of RSV in children aged ≤ 3 years increased significantly; this included both RSV-A and RSV-B in 2023, whereas in 2024, only that of RSV-B increased (Supplementary Fig. 1). Children aged ≤ 1 year are more susceptible to HPIV infection (*P* < 0.01), with HPIV-3 being the predominant type. In 2023, the positive rate of HMPV increased in children aged 2–3 years and ≥ 6 years (*P* < 0.01). By 2024, the detection rate of HMPV began to decline in children aged 2–3 years, a trend also noted in the 4–5 years age group. The positive rate of HAdV decreased, across all children (*P* < 0.05). FluA mainly infected children ≥ 6 years (*P* < 0.001), and HCoV-OC43 mainly infected children ≤ 5 years (*P* < 0.05). After the lifting of NPIs, the detection rates of FluB and HBoV showed little change across all age groups (*P* > 0.05) (Fig. 3).

### Pathogen seasonality

**Fig. 4:**
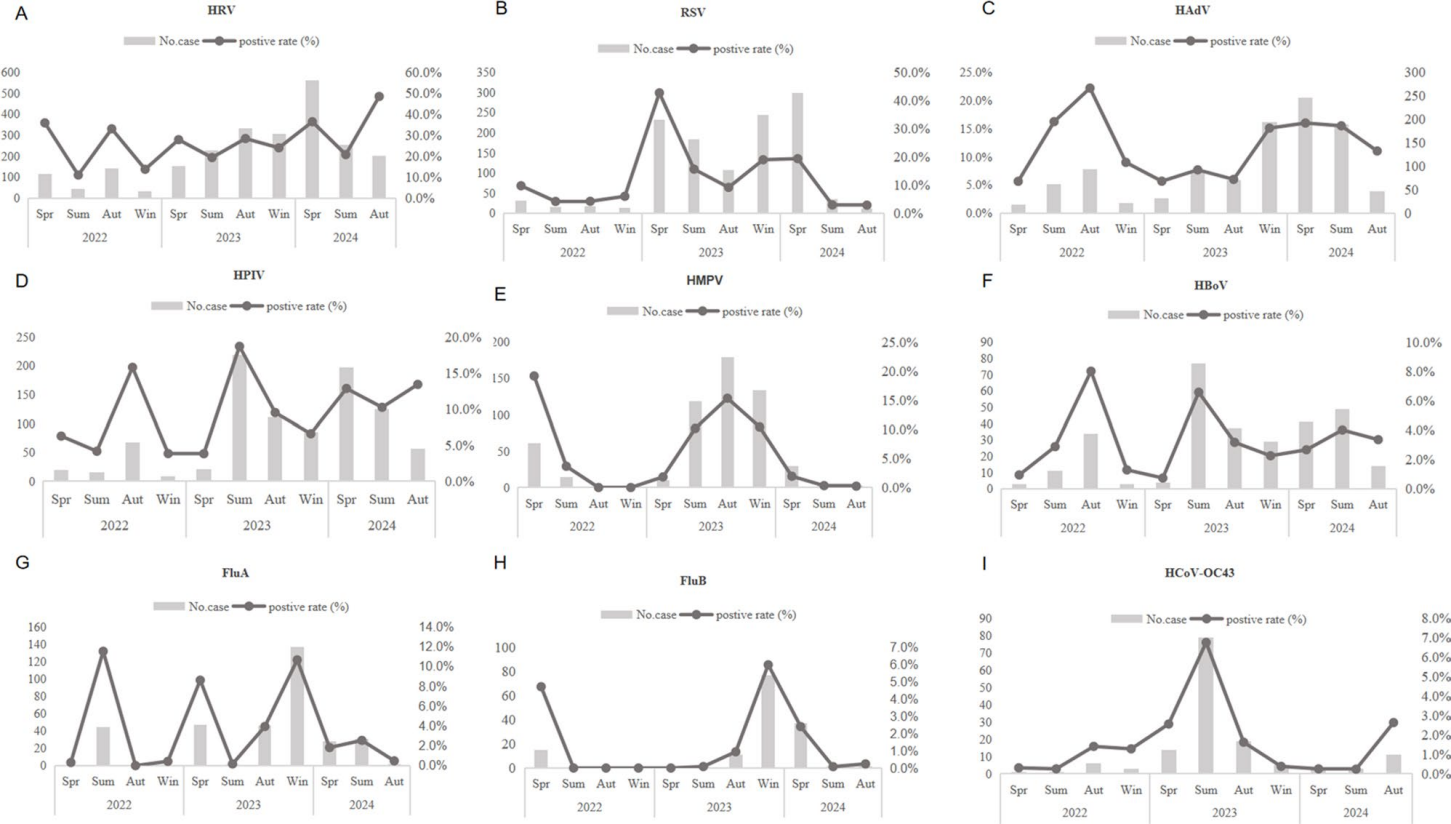
Seasonal distribution of pathogens from 2022 to 2024. The gray bars in the figure represent the number of positive samples for each pathogen, and the black lines indicate the positivity rate of the pathogens. **A**. Positive cases and infection rate of HRV. **B**. Positive cases and infection rate of RSV. **C**. Positive cases and infection rate of HAdV. **D.** Positive cases and infection rate of HPIV. **E**. Positive cases and infection rate of HMPV. **F**. Positive cases and infection rate of HBoV. **G**. Positive cases and infection rate of FluA. **H**. Positive cases and infection rate of FluB. **I.** Positive cases and infection rate of HCoV - OC43

We plotted the positivity rates of pathogens for different months in various periods, visualizing the positivity rates of pathogens to understand the seasonal variations of each pathogen. All NPIs were lifted in December 2022, however, no increase in the positive rate of RSV was observed in the winter of 2022, until the outbreak of RSV occurred in the spring of 2023, and RSV began to resume its normal seasonal cycle in the winter and spring of 2023–2024. Subtype analysis of RSV revealed that RSV-A was predominant in 2023, while RSV-B became the dominant subtype in 2024 (Supplementary Fig. 2). A similar epidemic pattern was observed for HMPV. Compared with 2022, HMPV showed an opposite epidemic pattern in 2023. There were no positive cases of HMPV in the autumn and winter of 2022. However, in 2023, HMPV emerged in the spring and maintained a high positivity rate starting from the summer, which continued through the winter of 2023. The positivity rate of HAdV did not increase in the early period after the removal of NPIs, but began to rise sharply in the winter of 2023, with the epidemic lasting until the summer of 2024. The epidemic peaks of HPIV and HBoV shifted from autumn 2022 to summer 2023, and similarly maintained high positivity rates in spring 2024. This characteristic was mainly observed in HPIV-3. Different types of HPIV have distinct seasonal patterns: HPIV-1 and HPIV-4 continued to circulate in autumn, while HPIV-2 was rarely detected in 2023, emerging instead in spring 2024 (Supplementary Fig. 2). In 2022, FluA was rarely detected except in the summer, it with a brief epidemic in the spring of 2023 and outbreaks in the autumn and winter of the same year. FluB exhibited a similar pattern, resuming its epidemic in the autumn and winter of 2023. HRV maintained a relatively high positive rate, but with little change across different seasons. HCoV-OC43 exhibited low positivity rates throughout 2022, with a transient surge in summer 2023 followed by a sharp decline in autumn. Subsequently, positivity rates remained low through 2024 (Fig. 4).

### Single infection, co-infection, and pathogen co-occurrence patterns

**Fig. 5:**
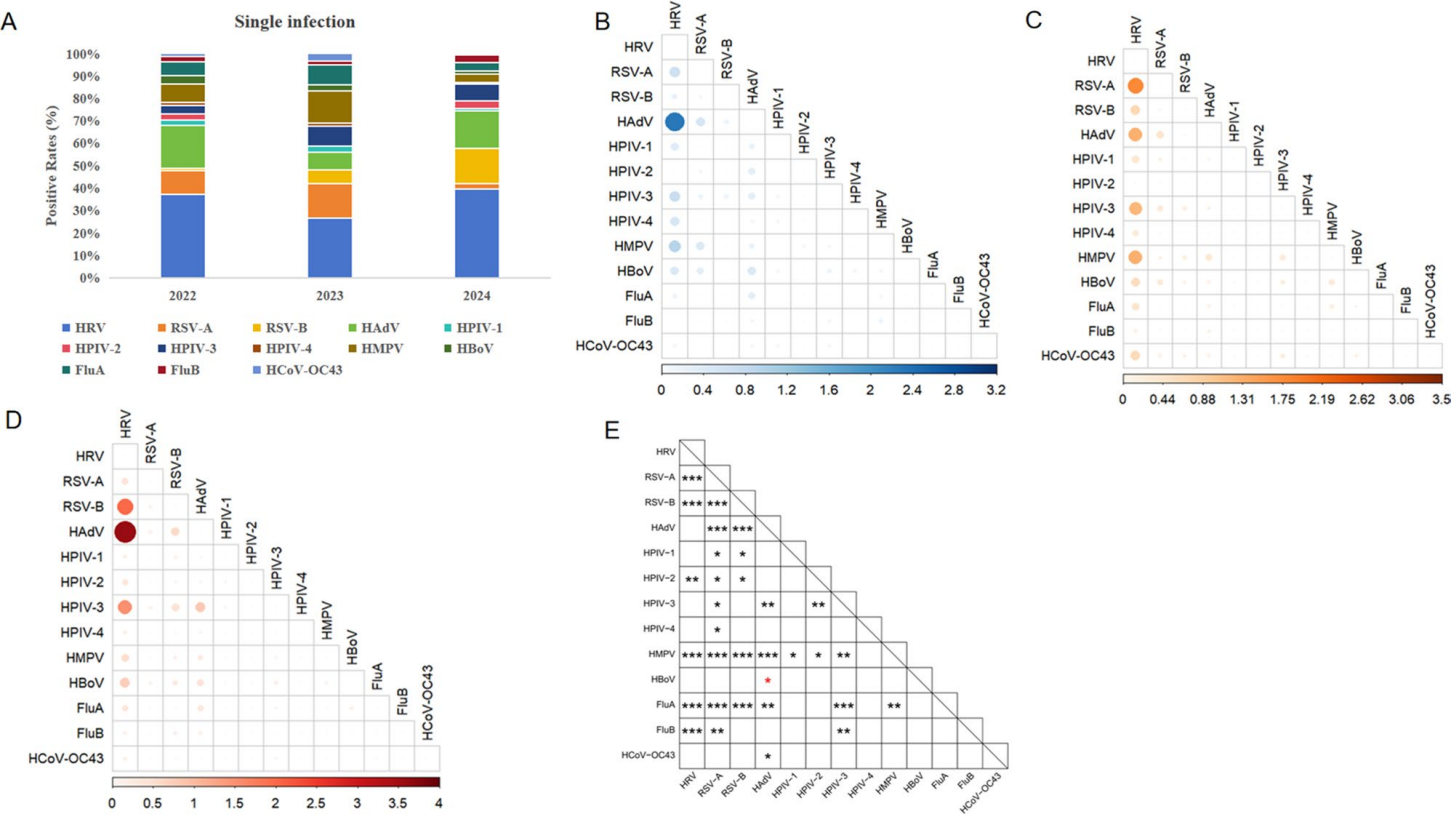
Infection patterns and interactions of pathogens from 2022 to 2024. **A**. Changes in the rate of single pathogen infections from 2022 to 2024. **B**, **C**, **D**. Represent the co-infection conditions of pathogens in 2022, 2023, and 2024, respectively. For pathogens “X” and “Y”, the numerator is the number of patients co - infected with “X” and “Y”, and the denominator is the total number of patients tested for both “X” and “Y”. Larger and darker circles indicate a higher co - infection rate between the two pathogens. **E**. Red * indicates co-occurrence with *p* < 0.05, while black * indicates negative co-occurrence with *p* < 0.05

Among the population seeking medical care, 620 children were infected with a single pathogen in 2022, with an infection rate of 44.5% (620/1392) (Table 1). HRV was the most common pathogen for single infections (16.5%), followed by HAdV (8.5%) (Fig. 5A). The co-infection rate was 11.9% (165/1392), with the most common co-infection being HRV + HAdV (2.3%) (Fig. 5B). In 2023, the single pathogen infection rate in children increased to 50.9% (1721/3382), significantly higher than in 2022 (*P* < 0.001). HRV, RSV-A and HMPV were the main pathogens for single infections, accounting for 13.6%, 7.8% and 7.3%, respectively. The co-infection rate rose to 15.0% (508/3382 ) (*P* < 0.01), with HRV + RSV-A being the dominant combinations (1.77%) (Fig. 5C). By 2024, both single and co-infections in children remained higher than 2022 (*P* < 0.01). The single infection rate was 49.4% (1975/4000), and the co-infection rate was 16.0% (638/4000). HRV (19.5%), HAdV (8.4%), and RSV-B (7.7%) are the main pathogens responsible for single infections. HRV and HAdV became the leading pathogens for co-infections (3.58%), followed by HRV + RSV-B and HRV + HPIV-3, accounting for 1.98% and 1.5%, respectively (Fig. 5D).

The co-occurrence analysis between pathogens revealed that only HBoV and HAdV exhibited a co-occurrence pattern. Moreover, no correlation (either positive or negative) were found between HBoV and other pathogens. Notably, RSV-A had negative co-occurrence patterns with most pathogens, including RSV - B, HAdV, HPIV-1, HPIV-2, HPIV-3, HPIV-4, HMPV, FluA, and FluB. In addition, a negative co-occurrence patterns was also observed between FluA and HMPV, and both of these viruses had negative co-occurrence patterns with other pathogens, including HRV, RSV-A, RSV-B, HAdV, and HPIV-3. Negative co-occurrence patterns involving HRV were also common, including with RSV-A, RSV-B, HPIV-2, HMPV, FluA, and FluB. Among different subtypes of the same virus, in addition to RSV, we also observed a negative co-occurrence patterns between HPIV-2 and HPIV-3, while no correlation was found between FluA and FluB (Fig. 5E).

### Restricted cubic splines reveal the relationship between age and the risk of pathogen infection

**Fig. 6:**
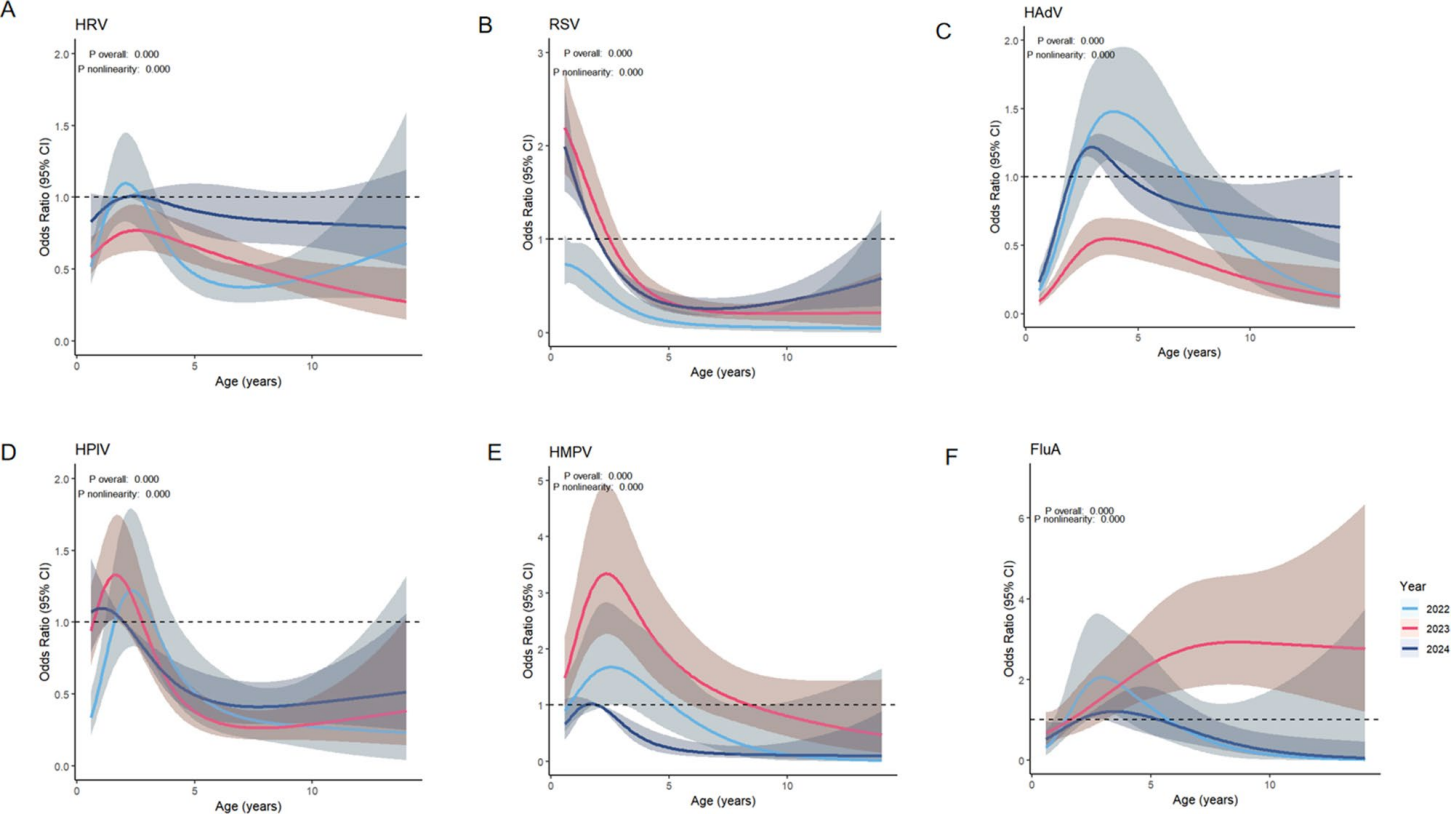
Analyzing the association between age and the risk of pathogen infection using Restricted Cubic Spline (RCS) analysis [14]. (**A**) HRV (**B**) RSV (**C**) HAdV (**D**) HPIV (**E**) HMPV (**F**) FluA

Furthermore, we employed restricted cubic splines (RCS) [14, 15] to analyze the nonlinear relationship between pathogen infection risk and age among children seeking medical care from 2022 to 2024, where an odds ratio (OR) >1 indicates a higher risk of infection. In 2022, children had a lower risk of RSV infection, while in 2023–2024, the risk of RSV infection increased among children under 3 years old. In 2022, children aged 4 had the highest risk of HAdV infection. In 2023, there was no high-risk population for HAdV infection, but in 2024, the risk of HAdV infection increased among children aged 2–3 years. In 2022, there was no statistically significant risk of HPIV infection among children. In 2023, children under 1 year old had an increased risk of HPIV infection, while the risk of HPIV infection decreased with age among children over 1 year old, stabilizing after the age of 5. In 2024, no high-risk population for HPIV infection was identified. We observed that in 2023, children under 2 years old had an increased risk of HMPV infection, which gradually decreased with age. In 2022, children aged 1–6 had a high risk of FluA infection. By 2023, children aged 1–14 had a high risk of FluA infection. The risk of HRV infection among children in all age groups was not statistically significant from 2022 to 2024 (Fig. 6).

In summary, our data indicate that after the cessation of the zero-COVID policy, the infection risk of certain pathogens changed among specific age groups of children seeking medical care. The risk of RSV infection increased among children under 3 years old, the risk of HPIV infection increased among children under 1 year old, the risk of HMPV infection increased among children under 2 years old, and the risk of FluA infection increased among children aged 1–14. In 2024, the risk of HAdV infection increased among children aged 2–3 years.

## Discussion

In December 2022, China announced the end of its dynamic zero - COVID policy and the complete lifting of NPIs [5]. In this study, we analyzed retrospective, multicenter data based on targeted next - generation sequencing results to understand the epidemiological changes in respiratory pathogens after the cessation of the zero-COVID-19 policy and complete relaxation of NPIs. A significant increase in pathogen positivity rates was observed in 2023 and 2024 after the lifting of NPIs compared to 2022, with an increase in the prevalence of children aged ≤ 1 year and ≤ 6 years. Our data show that the median age of children increased from 2 years during 2022–2023 to 3 years in 2024. This may be related to the restricted spread of viruses during the epidemic. After the zero - COVID policy was lifted, the increased opportunities for contact between groups led to an expansion of the age range of infected individuals. However, this change did not immediately appear in 2023, possibly due to the lag in the lifting of epidemic prevention and control measures in different regions. A study conducted at Shenzhen Children’s Hospital from 2020 to 2023 also found that the median age of children with RSV infection in 2023 was higher than that in 2020 and 2021, and this difference was statistically significant (*p* < 0.001) [16]. Older children are a group that needs attention in the post - epidemic period, which may mean that their susceptibility to certain pathogens has increased.

In 2022, when NPIs remained in effect, the low positivity rates of respiratory pathogens were unsurprising given their shared transmission modes with SARS-CoV-2. However, our primary focus lies in determining whether the epidemiological patterns of these respiratory pathogens will revert to pre-pandemic levels or persist in an altered phase following the complete relaxation of NPIs.

In previous years, high RSV positivity rates were detected in winter and spring across 16 Chinese provinces, including Hunan [17]. However, after NPIs were fully lifted in December 2022, no increase in RSV positivity rates was observed in the winter of 2022. Instead, an RSV outbreak occurred in spring 2023. In fact, during the pandemic, studies observed a decline in RSV antibody titers among young patients [18, 19], which could not explain the low - level detection of RSV in the early stages after the lifting of NPIs. A similar phenomenon was found in Beijing. The study suggests it may be linked to the local COVID-19 outbreak, which suppressed RSV transmission [20]. In addition, a low rate of co-infection between SARS - CoV − 2 and RSV has indeed been observed in multiple regions [21–23]. We also observed increased positivity rates of RSV - A and RSV - B in children under three, along with heightened infection risks in younger kids. This may stem from their weaker immunity. Similarly, Liu’s study found that in the post - pandemic era, RSV - infected children tend to suffer more severe symptoms [24].

HMPV showed a similar epidemiological change to RSV. From 2009 to 2021, HMPV was monitored to be prevalent in the winter and spring seasons [25], Until 2022, we observed that this epidemic pattern of HMPV was interrupted, with no cases of HMPV infection found in the autumn and winter seasons. However, with the relaxation of NPIs, HMPV began to emerge in the spring of 2023 and continued to be detected until the spring of 2024, with an off - season epidemic of HMPV occurring in the summer and autumn seasons. This resurgence pattern of RSV and HMPV indicates that these two pathogens may be more sensitive to the implementation and relaxation of NPIs. This sensitive phenomenon of HMPV has also been found in other countries. The NPIs implemented in Western Australia in early 2020 led to no HMPV - related positive cases admitted to the hospital within 12 months, followed by a significant surge in 2021, with the largest increase in admissions among children aged 1–4 years. These changes are related to population movement, herd immunity, and seasonality [26, 27]. Although HAdV can be continuously detected, no immediate changes were observed after the lifting of NPIs. It was not until the winter of 2023 that a rebound was observed, a phenomenon also observed in Ningbo [28]. It can be inferred that the seasonal changes of HAdV were not significantly affected by NPIs. In addition, HRV and HCoV - OC43 did not show typical seasonal changes.

In 2022, we only observed a high positive rate of HPIV in the autumn. After the lifting of NPIs, HPIV resumed its characteristic of being prevalent in the summer and autumn [29], with children under 1 year old becoming the main infected group, and HPIV maintained a high positive rate in the summer of 2024. The impact of NPIs on the epidemic pattern of HPIV varies by region. In Hainan, HPIV shifted from the winter and spring of 2021–2022 to the spring and summer of 2023 [30]. Notably, we also observed the same seasonal changes in HBoV as in HPIV, with the main epidemic peak in the summers of 2023 and 2024. In previous years, the infection peak of HBoV in Hunan was observed in the summer [31], suggesting that HPIV and HBoV may have begun to return to their pre - COVID − 19 pandemic seasonal characteristics. We also observed a slight rebound of HPIV and HBoV in the spring of 2024, a phenomenon also observed in Suzhou [32]. In 2022, we observed a significant decrease or even disappearance of FluA and FluB. In fact, in the early stages of the COVID − 19 pandemic, with the implementation of NPIs, a global decline in influenza viruses was monitored [33, 34], and from October 2020 to early 2021, the activity level of influenza viruses in the United States and Canada was at an all - time low [35, 36]. In 2023, we observed that the two pathogens resumed their typical seasonal characteristics in the autumn and winter. In 2023, the prevalence of FluA increased in children aged 6 years and older, which may be due to school - aged children returning to school and increased social activities after the pandemic stabilized. The seasonal pattern of influenza virus has been restored in Shenzhen and Hainan, with spring outbreaks being more typical [37, 38].

Our data indicate that co-infections between pathogens became more frequent after NPIs, increasing from 11.9% in 2022 to 15.0% in 2023 and 16.0% in 2024, with HRV being the main pathogen for co-infections from 2022 to 2024, indicating that preventive measures such as mask - wearing and NPIs had limited impact on the spread of HRV. After the lifting of control measures, the co - infection rates of RSV and HPIV increased the most. There may be several reasons for this phenomenon. First, during the pandemic, preventive measures such as mask - wearing and social distancing were not canceled, reducing people’s exposure to pathogens and leading to a lack of necessary stimulation for the immune system, thus making individuals more susceptible to multiple pathogens. Second, the increased prevalence of RSV and HPIV after the lifting of NPIs also increased the risk of co-infections.

Multiple pathogens exist in the human respiratory tract, and analyzing their co-occurrence or negative co-occurrence patterns can help understand potential epidemiological associations. Our study found that most viruses exhibited negative co-occurrence patterns, meaning they are less likely to infect simultaneously, especially in cases involving RSV - A. This finding may provide insights into the interactions between pathogens. The negative interaction between RSV and FluA has been demonstrated in studies, which can be explained by the type I interferon response induced by RSV - A in host cells that inhibits FluA infection [39]. Meanwhile, FluA can also inhibit RSV infection by increasing the expression of IFN - induced proteins (such as IFIT) [40]. On the other hand, we found negative co-occurrence patterns between influenza viruses and non-influenza viruses, including HRV, RSV-A, RSV-B, and HPIV-3, all of which have been reported to have negative interactions with influenza viruses [2, 41]. These associations may provide clues for future research into the interactions between viruses and their underlying mechanisms. It is worth noting that the co-occurrence patterns of pathogens are not only determined by the characteristics of the pathogens themselves but also by the host’s immune status and environmental factors. The relationship between these co-occurrence patterns and patient conditions is worth further exploration.

Our results indicated that younger children exhibited increased susceptibility to viral infections following the complete lifting of non-pharmaceutical interventions (NPIs), potentially attributable to their immature immune systems and reduced pathogen exposure during the pandemic. Many studies use the concept of “immune debt” to explain this phenomenon [42], which describes unintended negative consequences of prolonged infection suppression during pandemic control measures. It is worth noting that RSV infection risks remained elevated in younger children (under three years old) during both 2023 and 2024. A prospective observational study similarly reported higher frequency and severity of RSV infections in neonates post-pandemic compared to pre-pandemic and pandemic periods [43], underscoring this population’s heightened vulnerability during late-pandemic phases. In 2023, the population with a high risk of infection for FluA and HMPV increased, while in 2024, this range narrowed, suggesting potential recovery of population-level immunity by 2024. However, these dynamic patterns require ongoing surveillance to confirm.

This study has several limitations. First, although this is a multicenter, large - sample study, it was conducted within Hunan Province, and the results obtained may differ from those in other regions of China or other countries. Second, this is a retrospective study, relying on the completeness and accuracy of historical records, which may not represent all individuals seeking healthcare, and this approach makes it difficult to determine whether the observed epidemiological changes are entirely due to policy changes or influenced by other factors. Third, the retrospective design may introduce potential diagnostic bias. The confirmation of respiratory infection cases depends on clinical and laboratory data, which may be subject to inconsistencies in hospital records during retrospective collection. Another limitation of this study is the time scope of data collection. Our study period was from January 1, 2022, to October 31, 2024, so the data for 2024 only cover the first ten months. Since influenza activity typically peaks in the autumn and winter, our data may only represent the initial manifestation of influenza viruses after the termination of the zero - COVID policy. The seasonal variations of influenza should be interpreted with caution. Further research is needed to better understand the epidemiological changes in 2024 and beyond. Moreover, in 2022, during the implementation of the zero - COVID policy, people’s healthcare - seeking behaviors changed. Some individuals with respiratory infection symptoms might choose to self - treat at home rather than visit the hospital. However, after the zero - COVID policy was lifted, the number of patients visiting the hospital gradually returned to normal. Therefore, although we did not change the inclusion criteria of this study, there was a gradual increase in the sample size during 2023–2024. The increase in sample size may make the individual differences within the study population more pronounced, thus becoming a potential confounding factor. Additionally, since this study included children who sought medical care at the hospital and selected tNGS as the detection method, as we mentioned, the choice of this method usually meets specific criteria. This may lead to results that are biased towards the pathogen prevalence characteristics of children with severe illnesses, and may not reflect the conditions of children with milder symptoms or those who did not seek medical care. The positivity rate of HBoV, FluB, and HBoV-OC43 is low in older children. When the sample size is insufficient to support the effective distribution of outcome events, even the use of flexible non-linear modeling methods such as RCS can easily lead to fitting bias. The results lack statistical power and clinical reference value. We need to expand the sample size or explore more robust analysis strategies for small samples, such as Bayesian methods, in the future to more comprehensively reveal the interactive effects of age and time on infection risk.

To address these limitations, it is necessary to consider expanding the geographical scope and conducting prospective studies in the future, in order to enhance the general applicability of the research findings and reduce the impact of diagnostic bias and confounding factors.

## Conclusions

In summary, as epidemic prevention and control measures are gradually relaxed, the reduced frequency of mask - wearing and increased outdoor activities have affected the transmission of respiratory pathogens. Clinically, it is necessary to monitor the epidemiological situation of common respiratory pathogens in children after the relaxation of COVID − 19 prevention and control measures, enhance children’s immunity, and maintain continuous monitoring for timely treatment.

## Supplementary Information

Below is the link to the electronic supplementary material.

Supplementary Material 1

Supplementary Material 2

## Data Availability

The datasets of this study can be obtained from the corresponding author upon reasonable request.

## Abbreviations

ARTIs: Acute respiratory tract infections
RSV: Respiratory syncytial virus
RSV-A: Respiratory syncytial virus type A
RSV-B: Respiratory syncytial virus type B
HRV: Human rhinovirus
HAdV: Human adenovirus
HPIV: Human parainfluenza virus
HPIV-1: Human parainfluenzavirus type 1
HPIV-2: Human parainfluenzavirus type 2
HPIV-3: Human parainfluenzavirus type 3
HPIV-4: Human parainfluenzavirus type 4
HMPV: Human metapneumovirus
HBoV: Human bocavirus
FluA: Influenza A virus
FluB: Influenza B virus
HCoV-OC43: Human coronavirus OC43
NPIs: Non-pharmaceutical interventions
tNGS: Targeted next-generation sequencing
