# Supplementary material for: Epidemiological analysis of pediatric respiratory pathogens in Hunan, China: a retrospective multicenter study from 2022 to 2024
Source: BMC Infect Dis. 2025 Dec 10;26:60. doi: 10.1186/s12879-025-12283-6 (PMC12801765; doi:10.1186/s12879-025-12283-6)
Supplement: Supplementary file 1 — Supplementary Material 1 [file 12879_2025_12283_MOESM1_ESM.docx]

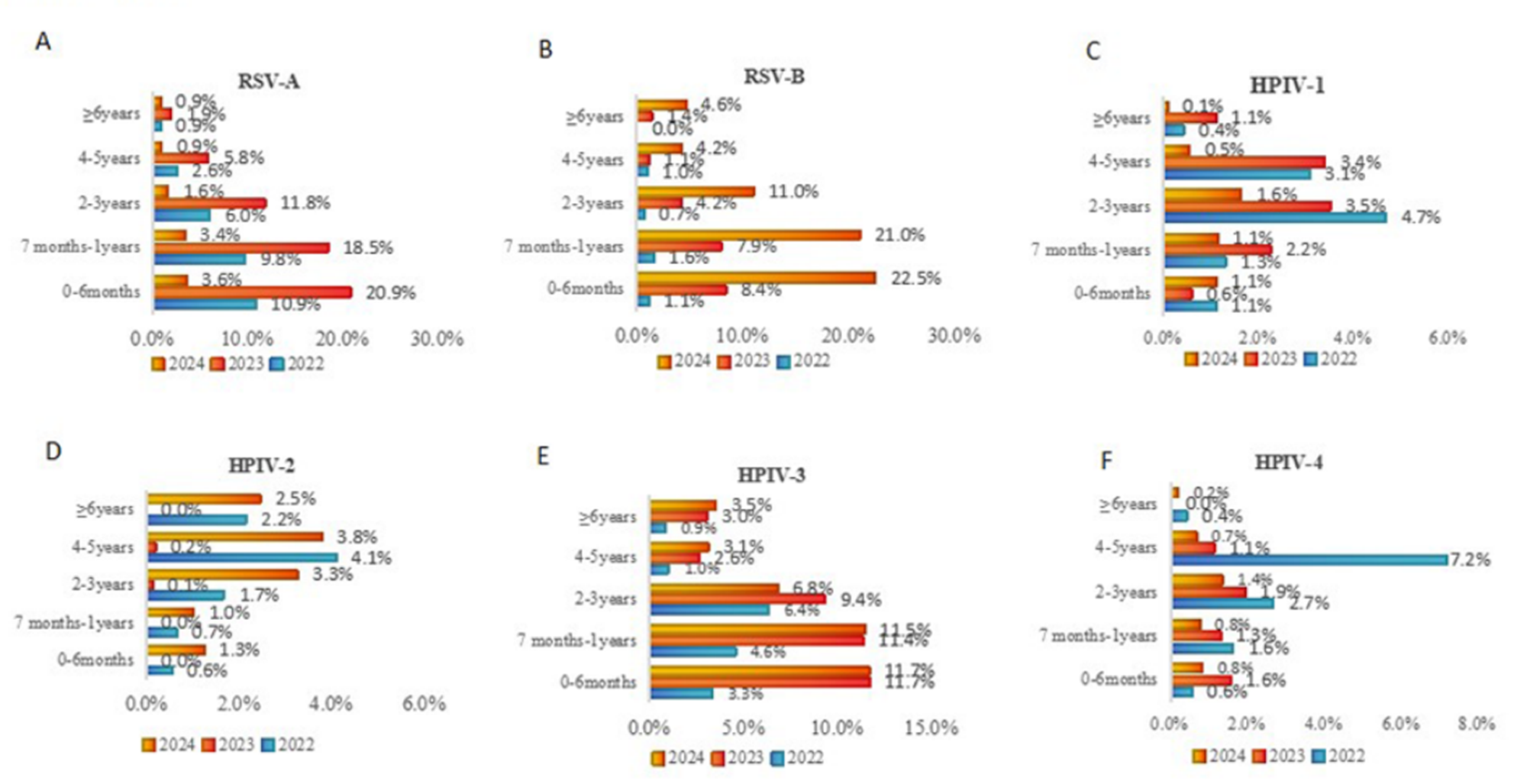


**Supplementary Fig1.** Epidemiological characteristics of RSV and HPIV subtypes in different age groups from 2022 to 2024. A. RSV-A B. RSV-B C. HPIV-1 D. HPIV-2 E. HPIV-3 F. HPIV-4
