# Supplementary material for: Epidemiological analysis of pediatric respiratory pathogens in Hunan, China: a retrospective multicenter study from 2022 to 2024
Source: BMC Infect Dis. 2025 Dec 10;26:60. doi: 10.1186/s12879-025-12283-6 (PMC12801765; doi:10.1186/s12879-025-12283-6)
Supplement: Supplementary file 2 — Supplementary Material 2 [file 12879_2025_12283_MOESM2_ESM.docx]

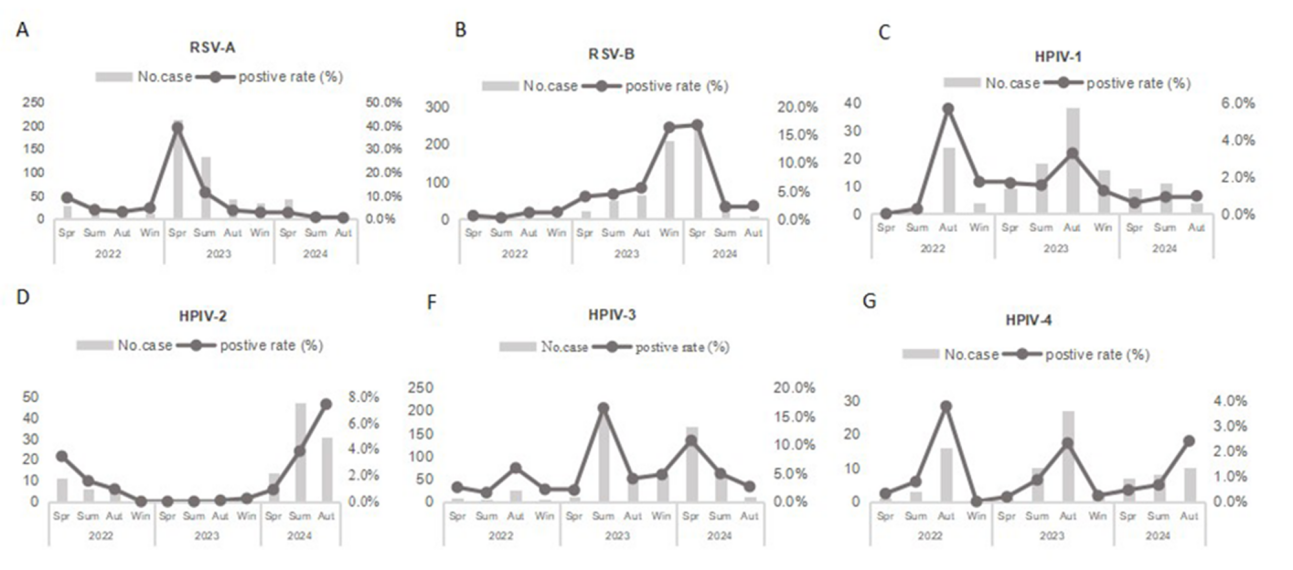


**Supplementary Fig2.** Seasonal distribution characteristics of RSV and HPIV subtypes from 2022 to 2024. The gray bars in the figure represent the number of positive samples for each pathogen, and the black lines indicate the positivity rate of the pathogens. **A.** Positive cases and infection rate of RSV-A **B.** Positive cases and infection rate of RSV-B **C.** Positive cases and infection rate of HPIV-1 **D.** Positive cases and infection rate of HPIV-2 **E.** Positive cases and infection rate of HPIV-3 **F.** Positive cases and infection rate of HPIV-4
